# 2D Chitosan-Based Films: A Proteomic Mass Spectrometry Study of Chondrocyte Phenotype as a Function of Cell–Biomaterial Interactions

**DOI:** 10.3390/ijms262110291

**Published:** 2025-10-22

**Authors:** Alessandro Zaccarelli, Roberta Saleri, Elena De Angelis, Francesca Ravanetti, Attilio Corradi, Paolo Borghetti

**Affiliations:** 1 Food and Drug Department, University of Parma, Viale delle Scienze 17/a, 43124 Parma, Italy; alessandro.zaccarelli@unipr.it; 2 Department of Veterinary Science, University of Parma, Strada del Taglio 10, 43126 Parma, Italy; roberta.saleri@unipr.it (R.S.); elena.deangelis@unipr.it (E.D.A.); attilio.corradi@unipr.it (A.C.); paolo.borghetti@unipr.it (P.B.)

**Keywords:** 2Dimensional culture, chitosan, chondrocyte dedifferentiation, hyaluronic acid, LC-MS/MS proteomic analysis, tissue engineering

## Abstract

In vitro chondrocyte expansion is key to all tissue engineering (TE) strategies using adult differentiated articular chondrocytes. Unfortunately, high proliferation rates in vitro can cause a progressive loss of chondrocyte phenotype (dedifferentiation) during culture passages. This can impair the quality of newly formed tissue after implantation because dedifferentiated chondrocytes mainly produce fibrocartilage, which hinders successful cartilage repair. Freshly isolated chondrocytes from equine articular cartilage were grown as a primary culture on tissue culture dishes and on 2D chitosan or chitosan/hyaluronic acid films. To evaluate chondrocyte differentiation during in vitro expansion, morphological observations, gene expression of chondrocyte phenotype markers, and LC-MS/MS shotgun proteomics were performed. All types of 2D cultures showed significantly reduced differentiation compared with freshly isolated cells, but chondrocytes grown on biomaterials maintained a rounded morphology and the gene expression of differentiation markers. Interestingly, pairwise proteomics comparison revealed a remarkable number of differentially expressed proteins, highlighting the different dynamics occurring in each experimental condition at the protein level. Based on novel insights into differentiation-dedifferentiation mechanisms, hypotheses were generated to explore new markers implicated in dedifferentiation and the role of biomaterials in this process by investigating the biological pathways associated with the reduced phenotype.

## 1. Introduction

Articular cartilage (AC) is a hyaline connective tissue constituted by a highly hydrated extracellular matrix (ECM) with specific mechanical properties. Articular chondrocytes are highly specialized cells that are embedded into the ECM and are responsible for its synthesis and degradation.

The chondron is the functional unit of cartilage. It consists of a chondrocyte, located within its lacuna and surrounded by the pericellular matrix (PCM), which mediates interactions between the chondrocyte and the surrounding extracellular matrix (ECM), consisting of collagen type II and LAPs (large aggregating proteoglycans). Within its PCM, the chondrocyte primarily exhibits a round morphology, which is associated with its specialized functions in articular cartilage homeostasis (i.e., its differentiated phenotype) [1].

In both human and veterinary patients, articular cartilage damage and the onset and progression of osteoarthritis (OA) are the leading causes of joint pain and disability. Successful treatment is a major challenge for orthopedic patients because articular cartilage has a very poor capacity for spontaneous repair [2,3]. This has led to the formation of fibrocartilaginous tissue without the same mechanical properties as native cartilage [4]. Therapies including anti-inflammatory drugs and intra-articular hyaluronate injections to relieve pain are not effective at slowing the progression of the disease. One option for treating cartilage damage is surgery including cell-based therapies using biomaterial scaffolds. The latter approach involves the isolation, in vitro expansion, and culture of primary or precursor cells in a biocompatible matrix for subsequent in vivo implantation into the damaged tissue. When cell-based therapies use differentiated chondrocytes isolated from articular cartilage, a limiting factor for a successful repair is the maintenance of the mature and functional chondrocyte phenotype in vitro. Thus, to have an optimal cell number for implantation, in vitro expansion is needed. The problem arises when a progressive loss of chondrocyte differentiation (dedifferentiation) occurs during serial culture passages. Chondrocyte dedifferentiation is characterized by a reduced expression in the differentiating markers of mature chondrocytes (collagen type II, aggrecan, SOX9) and an increase in dedifferentiated markers (COL1 and RUNX2). It is also associated with the switch from a roundish morphology to a fibroblast-like shape [5]. Therefore, the maintenance of chondrocyte differentiation and/or the reduction in dedifferentiation is a crucial prerequisite when adult chondrocytes are used for cartilage repair [6,7]. Research is attempting to increase new or more complete strategies to maintain chondrocyte differentiation or allow their redifferentiation [8,9], studying novel supports that maintain chondrocyte differentiation and ECM production. The choice of biomaterial is thus critical for the success of such tissue engineering approaches. Much effort has been made to develop new substitutes that mimic cartilage structure [10,11].

Some natural (e.g., silk, fibrin, collagen, chitosan) and synthetic polymers (e.g., PLA, PGA) have been tested as biomaterials for tissue engineering to improve chondrocyte phenotype and differentiation [12]. Chitosan, a derivative of the natural polysaccharide chitin, is a widely used biomaterial due to its biocompatibility, biodegradability, non-toxicity and low cost [13]. It has been used in numerous applications such as drug delivery [14,15], wound healing [16,17], as a chelating agent in membrane filters for water treatment [18], and in tissue engineering [19,20]. Chitosan has a structure similar to glycosaminoglycans (GAGs), structural components of native cartilage ECM. For this reason, it has been indicated as a suitable biomaterial for the growth and functional maintenance of human chondrocytes in in vitro cultures [21,22,23,24]. Hyaluronate is a glycosaminoglycan, a natural component of the cartilage ECM and of the synovial fluid of joints. Hyaluronate acts as an environmental cue to regulate cell behavior during embryonic development, healing processes, and inflammation [25,26].

In the present work, expanded articular chondrocytes were used due to their inability to maintain a differentiated state when cultured in adhesion. Therefore, they represent a useful in vitro model to evaluate innovative substrates to maintain differentiation or reduce dedifferentiation [6,27]. Specifically, we chose to use chondrocytes at the first culture passage (P1). This is the early expansion phase when the most critical mechanisms of dedifferentiation occur [28]. In our research, chondrocytes, isolated from equine articular cartilage, were expanded in vitro in adhesion to tissue culture dishes and to chitosan or chitosan/hyaluronic acid films. The focus was on the cellular changes that occur during this process. To this end, we analyzed chondrocyte behavior at the gene and protein expression level to assess how biomaterials influence cellular changes during in vitro expansion.

Here, 2D films were prepared with a concentration of chitosan and a thickness that allowed the cells to be in contact and influenced by the biomaterial. The addition of hyaluronic acid makes this thermoplastic biomaterial much more suitable for chondrocyte cell culture [29]. Morphological observations and the gene expression of chondrocyte markers were evaluated. In addition, a shotgun proteomic approach was used to elucidate the modulation of chondrocyte differentiation during in vitro expansion to improve the knowledge of specific protein events responsible for chondrocyte dedifferentiation mechanisms.

## 2. Results

### 2.1. Effects of Biomaterials on Cell Morphology and Expression of Chondrogenesis Gene Markers

**Figure 1:**
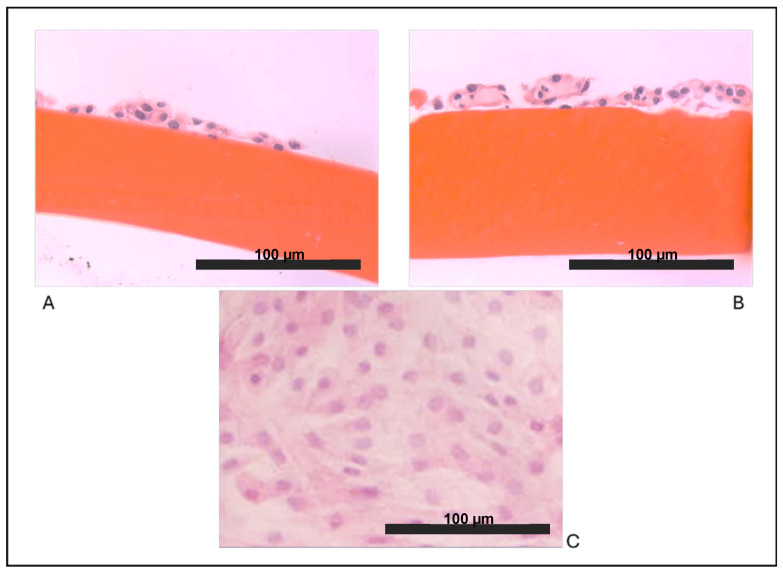
Optical microscopy observations: (**A**) chondrocytes grown on a film of chitosan (F), (**B**) chondrocytes grown on chitosan/hyaluronic acid (F+HA) films (hematoxylin/eosin stain, 40×), and (**C**) chondrocytes grown on a tissue culture dish (10×).

As expected, we observed that articular chondrocytes, grown in vitro in adhesion on plastic (Figure 1C) assumed a fibroblast-like morphology and within 2 weeks, they formed a monolayer of flattened cells, suggesting that dedifferentiation occurred early. Our results showed that both cultures on biomaterials allowed the maintenance of the chondrocyte round-like morphology within the two weeks of culture in vitro. Chondrocytes cultured on chitosan film (Figure 1A) and on chitosan film with HA (Figure 1B) showed a remarkable ability to form aggregates of spherical chondrocytes, as seen in vivo.

**Figure 2:**
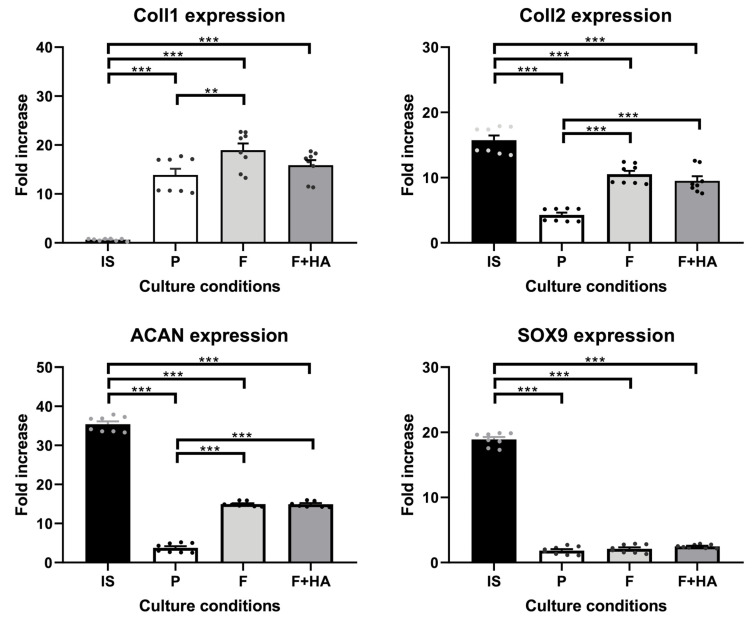
Gene expression of *Coll1*, *Coll2*, *Acan*, and *Sox9* in chondrocytes freshly isolated (IS), cultured in adhesion on a tissue culture dish (P), on chitosan film (F), and on chitosan film+hyaluronic acid (F+HA) for 2 weeks. **: *p* value < 0.01; ***: *p* value < 0.001. Data are expressed as the mean ± standard deviation, calculated on three technical replicates. The plot shows significant brackets to specify multiple comparisons between groups (one-way ANOVA followed by a Dunnett’s post hoc test).

Furthermore, these morphological findings were partially supported by the expression levels of the molecular markers involved in the differentiation process. The gene expression of relevant markers of chondrocyte phenotype was evaluated as described in Section 4.7. Supplementary Figures S2 and S3 show the expression profiles of all genes analyzed. Figure 2 focuses on key markers of the chondrocyte phenotype. The genes included in Figure 2 were also selected to align with the proteomic analysis, allowing for a consistent comparison between the transcriptomic and proteomic data. As shown in Figure 2, *Coll2*, *Acan*, and *Sox9* were strongly decreased in chondrocytes grown on tissue culture dishes (P) in relation to freshly isolated chondrocytes (IS), while *Coll1* gene expression was significantly increased. Chondrocytes cultured on both types of films showed higher *Coll2* and *Acan* gene expression compared with P but lower compared with IS. In addition, the gene expression of *Coll1* was significantly increased, and the gene expression of *Sox9* was significantly decreased in chondrocytes grown on both types of films when compared with IS.

As above-mentioned, the gene expression of other markers was evaluated. The expression of ECM markers such as collagen type VI (*Coll6*) and *COMP* was upregulated in cells cultured on biomaterials compared with P (Supplementary Figure S2). The expression of transcription factors associated with chondrocyte phenotype (in addition to *Sox9*) was also assessed. The gene expression of *RUNX2*, a marker of dedifferentiation, was significantly increased in P but decreased in F and especially in F+HA (Supplementary Figure S3). Considering the information obtained from the morphological and gene expression analysis, a liquid chromatography-mass spectrometry (LC-MS/MS) shotgun proteomic analysis was performed to generate a first hypothesis of the biochemical mechanisms occurring at the cellular level.

### 2.2. Exploratory Data Analysis of Differentially Expressed Proteins

**Figure 3:**
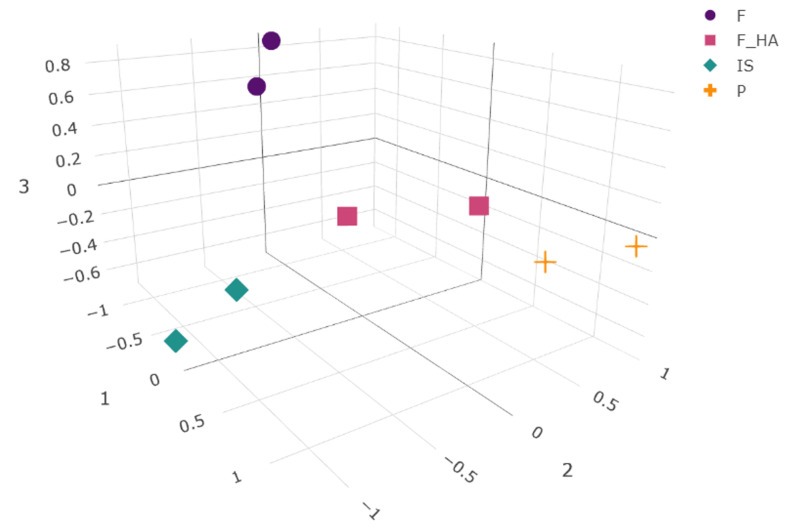
Three-dimensional embedding obtained by the UMAP algorithm. Normalized peak areas from all of the identified and filtered proteins in each experimental condition (as biological replicates) were used for calculation. The chondrocyte samples were labeled as follows: freshly isolated (IS), cultured in adhesion on a tissue culture dish (P), cultured on a chitosan film (F), and cultured on a chitosan film with hyaluronic acid (F+HA).

To our knowledge, this work represents one of the first proteomic analysis applied to study the behavior of primary chondrocytes during the first expansion phase (P1) in vitro when cultured in adhesion on tissue culture dishes (P) or on bidimensional films of chitosan (F) and chitosan/hyaluronic acid (F+HA). Freshly isolated chondrocytes (P0) and first in vitro passage chondrocytes (P1) cultured on a conventional culture system (in adherence to tissue culture dishes) were selected as references of the differentiated and early dedifferentiated states, respectively. To characterize the changes in the global protein expression of the cell proteome in response to biomaterials, a label-free quantitative proteomic approach was applied. As a result, 4865 proteins were identified in all samples. The differential expression of the identified proteins was calculated as described in Section 4.10. This resulted in a high number of differentially expressed proteins (DEPs) within the different conditions (2610, 2587, 2505, and 1870 for IS, F, P, and F+HA, respectively). The overall results of the pairwise comparisons are shown in the volcano plots in Supplementary Figure S4. The normalized peak areas of all of the identified proteins (filtered as described in Section 4.10) were used to evaluate the presence of patterns in the global protein profile between conditions. UMAP was used to calculate the distances between points, and the projections of the conditions as points in a low-dimensional space are shown in Figure 3.

As a nonlinear dimension reduction algorithm, UMAP was used because of its ability to preserve both the global and local structures thanks to the combination of Riemannian geometry and algebraic topology. By highlighting patterns derived from protein abundance within the samples, the algorithm was able to project the four conditions into different positions within the embedding space, reflecting the overall high number of significant DEPs resulting from pairwise comparisons. As expected, the semi-quantitative protein profile of IS and P resulted in separate clusters, suggesting a different protein expression of the cells during the P0–P1 passage. Interestingly, F and F+HA also clustered separately, indicating a different cellular response to the biomaterials. Looking at these two clusters, F+HA was in a centered position compared with the others, resembling an intermediate state with common features among the experimental conditions. Although F shared the second and the third UMAP coordinates with IS, its low-dimensional projection was quite distinct. Dimensionality reduction and data visualization allowed us to find interesting clues necessary to build a hypothesis about the mechanisms involved in differentiation and dedifferentiation.

### 2.3. Data Interpretation and Correlation with Chondrocyte Phenotype Protein Markers

**Figure 4:**
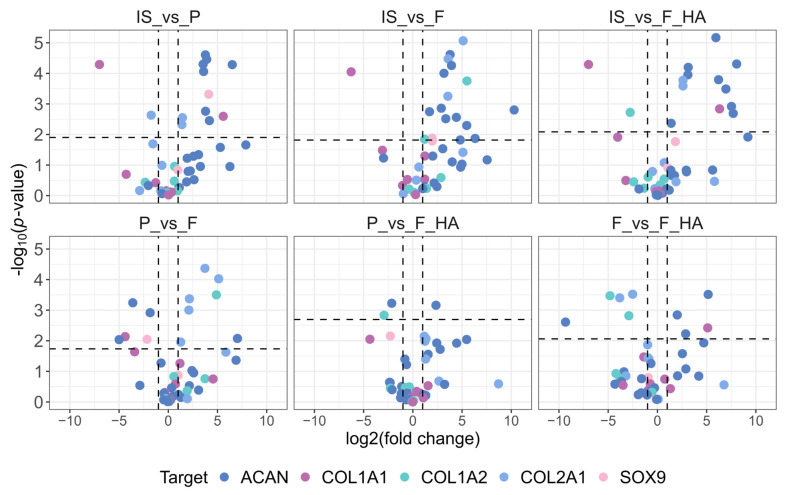
Fold change and statistical significance of chondrocyte phenotype protein markers in pairwise comparison. Proteins are represented by all of the precursor peptides identified by shotgun proteomics. The chondrocyte samples were labeled as follows: freshly isolated (IS), cultured in adhesion on a tissue culture dish (P), cultured on a chitosan film (F), and cultured on a chitosan film with hyaluronic acid (F+HA).

For comparison with the gene expression data, we first evaluated the protein levels of COL1 (A1 and A2), COL2, ACAN, and SOX9. In addition, we analyzed proteins involved in actin organization (CFL (1 and 2), ARPC2, SCIN), cell adhesion (FN1, ITGA5, ITGA3, ITGB1), and cytoskeletal-nuclear remodeling (LMNB1, CDC42). For clarity, the biomarkers were grouped into three graphical representations. Volcano plots illustrating the differential expression of the first set of biomarkers are shown in Figure 4, while pairwise comparisons of actin organization, cell adhesion, and cytoskeletal-nuclear remodeling proteins are shown in Supplementary Figures S5–S7.

As shown in Figure 4, the expression of cartilage functional markers such as COL2A1, ACAN, and SOX9 decreased with increasing passage number (P0–P1). This indicates an early and strong reduction in the functional phenotype, as expected. The dedifferentiation process at P1 (P, F, F+HA conditions) was also highlighted by the increased expression of dedifferentiation related genes such as COL1A1. When comparing the biomaterials (F, F+HA) and tissue culture dish (P) culture conditions, the expression of ACAN and SOX9 was shifted toward F in the P–F comparison, whereas P and F+HA appeared to be similar. COL2A1 expression was higher in P and F+HA compared with F. Regarding actin organization, the expression of cofilin (CLF 1/2) and actin related protein 2/3 (ARPC2) was examined in all experimental conditions. These proteins could contribute to the decrease in G/F-actin ratio associated with the loss of chondrocyte phenotype. Therefore, CFL 1/2 and ARPC2 were overexpressed in P (Supplementary Figure S5). Cells grown on biomaterials were found to express lower levels of these proteins, particularly for F. Adseverin (SCIN) was overexpressed only in IS. Cell adhesion proteins play a key role in mediating cell–ECM interactions. Fibronectin (FN1) and integrin α3β1 (ITGA3-ITGB1) were mainly overexpressed in P. Integrin α5 was downregulated in all conditions except IS (Supplementary Figure S6).

Finally, the expression of structural remodeling markers was evaluated. Lamin B1 and the Rho GTPase CDC42 were both overexpressed in P. The expression levels of these markers were downregulated in the other conditions, especially in IS and F (Supplementary Figure S7).

**Figure 5:**
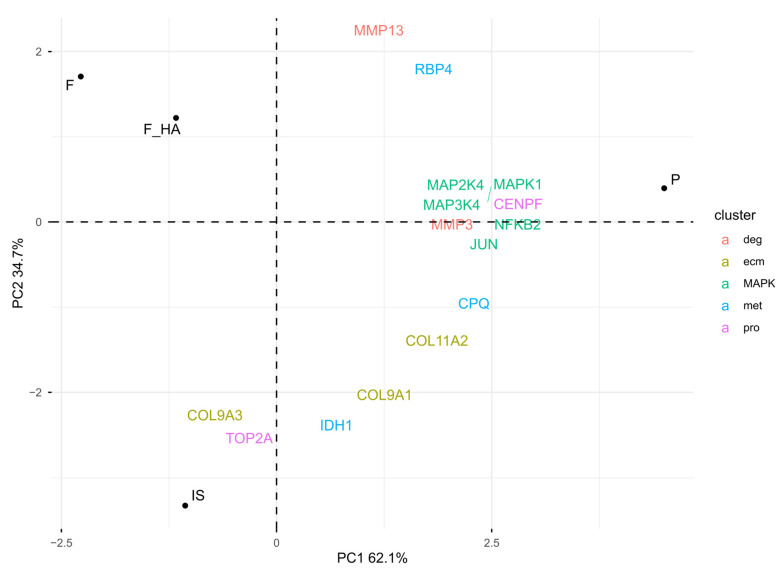
PCA biplot: visualization of the chondrocyte phenotype and MAPK pathway biomarkers in relative comparison between culture conditions (normalized LC-MS/MS peak areas were averaged between technical replicates). The chondrocyte samples were labeled as follows: freshly isolated (IS), cultured in adhesion on a tissue culture dish (P), cultured on a chitosan film (F), and cultured on a chitosan film with hyaluronic acid (F+HA).

Overall, these observations emphasized dedifferentiation in P, while biomaterials appeared to reduce phenotype loss. To understand the biological mechanisms behind the positive influence on chondrocyte phenotype by F and F+HA, proteins associated with different stages of chondrocyte dedifferentiation were examined (see Section 4.10). Clusters 1 (pro) and 2 (ecm) were characterized by proliferative genes and cartilage extracellular matrix genes, respectively. Cluster 3 (met) was represented by metabolic-related genes, while cluster 4 (deg) was described by degradation-related genes such as metalloendopeptidase. In addition, the metabolic stress associated with early dedifferentiation was evaluated. The focus was on the MAP kinase (MAPK) pathway. Figure 5 shows the PCA biplot calculated using the protein level of cluster-related proteins and MAPK pathway biomarkers within the experimental conditions.

As shown by the PCA biplot, the expression of specific markers was consistent with the chondrocyte phenotype signature. IS was mostly characterized by the pro and ecm markers, while the direction of met-related proteins, moving from IS to P, resembled the early stage of phenotype loss. Consistent with this observation was the higher expression of metalloendopeptidases (MMP3, MMP13), MAPK proteins (MAPK1, MAPK2K4, MAPK3K4), and the corresponding nuclear responsive proteins that mediate the downstream reaction (JUN, NFKB2) in P. F and F+HA were separated from the other condition, suggesting a different clusterization that could not be described by cluster-specific markers of cell passage. Interestingly, despite being P1 cells, the expression of MAPK pathway markers within F and F+HA was downregulated compared with P, suggesting a possible reduction in ROS. These findings may bridge the gap between the morphological form of P1 cells cultured on biomaterials and the lack of evidence for the biological mechanisms responsible. Finally, GO enrichment analysis was performed on the overexpressed proteins under all experimental conditions (see Supplementary Tables S7–S10). Supplementary Figure S8 shows the ten most statistically significant over- and underrepresented proteins in F and F+HA. These results are discussed in the next section.

## 3. Discussion

Chondrocyte expansion in vitro is required when adult articular chondrocytes are used for cell-based therapy applied for cartilage regeneration; unfortunately, this increased proliferation can progressively cause a loss of their differentiation and negatively affect the success of cell transplantation in vivo. The susceptibility of isolated chondrocytes to the differentiation/dedifferentiation process in monolayer cultures [30] is translated not only in a morphological change (from roundish to fibroblast-like morphology) [31], but also in a switch of the extracellular matrix components produced. As cell morphology is critical for cell function [32], the surrounding ECM produced by the chondrocytes themselves plays a role in maintaining their differentiated phenotype [33]. Numerous biomaterials are being tested to improve the maintenance of chondrocyte differentiation, and then the choice of biomaterial is critical to the success of tissue engineering approaches to cartilage repair [34].

The present study aimed at enhancing the knowledge of adult chondrocyte behavior during in vitro dedifferentiation. In addition, it focused on the cellular response to biomaterials, in particular how cell interactions with chitosan and chitosan/hyaluronic acid can modulate this process.

Chitosan structure is similar to glycosaminoglycans (GAGs) found in vivo in cartilage ECM, and studies have shown that chitosan hydrogel mimics the physiological environment populated by chondrocytes in vivo, which is characterized by gaps surrounded by extracellular matrix, low oxygen levels, and nutrient concentrations [35]. In addition, hyaluronic acid has been shown to support cell proliferation and maintain the chondrogenic phenotype [36].

We can hypothesize that the maintenance of a round-like morphology and the remarkable ability to form aggregates of spherical chondrocytes, observed in morphological images of cells cultured on chitosan films, was initially due to cell–chitosan interactions, and subsequently maintained by cell–cell interactions favored by the biomaterial [37]. The addition of HA favors the same effect, suggesting hyaluronic acid as a good additive component for biomaterials.

These considerations were supported by the gene expression analysis. Although the expression level of the dedifferentiated cell marker collagen type I (*Coll1*) was not attenuated by the culture on both type of films, the gene expression of typical cartilage ECM markers, such as *Acan*, *Coll2*, *coll6*, and *COMP*, was improved by the culture on chitosan and chitosan+HA films. *Sox9* expression was not altered.

Notably, higher *coll6* gene expression in chondrocytes grown on both biomaterial films compared with P can initiate the rebuilding of the pericellular matrix (PCM). This small collagen is a primary component of the matrix that surrounds the chondrocyte. It plays a role in the integrity of the chondron and in cell interaction with the extracellular matrix (ECM). In fact, type VI collagen expression diminishes during chondrocyte dedifferentiation and is restored during redifferentiation [1].

Furthermore, the increased cell survival of chondrons is associated with collagen VI and certain stress proteins (HSP70) [38].

In addition, COMP is another marker of chondrocyte differentiation and is a crucial protein in the overall cartilage ECM, specifically located in the territorial and interterritorial matrices, where it interacts with collagen type II, glycoproteins, and proteoglycans [39].

The gene expression of *RUNX2* was attenuated in the culture on both types of film used. The gene expression of *ALK5* and *SMAD2* was also upregulated in F and F+HA compared with P. These two genes belong to the same signaling pathway, which has important anti-hypertrophic and chondrogenic effects, contributing to the maintenance of ECM integrity [40].

Overall, these results suggest that biomaterials only partially attenuated the dedifferentiation process at the mRNA level during cell expansion adherent culture. However, cells growing on biomaterial films exhibited the rounded shape characteristic of mature chondrocytes.

Consistent with these observations, pseudotime analysis of the transcriptome showed that differentiation markers such as COL2A1 and ACAN are immediately downregulated during early dedifferentiation [41]. Nevertheless, the cells growing on the biomaterial films had the rounded shape typical of mature chondrocytes. The proteomic profile of P0–P1 cells was also examined to identify differentially expressed proteins. This information was used to generate a first hypothesis about the biological mechanisms associated with the phenotypic changes. Although shotgun proteomics is not a gold standard as a precise and reproducible quantitative method [42], the amount and quality of information that can be extracted from exploratory data analysis is essential for hypothesis-driven investigations.

The results from the proteomic analysis exhibited a good correlation with those derived from the qPCR. However, the degree of correspondence across the two datasets was not completely consistent. This discrepancy underscores the fact that the abundance of transcripts does not invariably correlate with the protein levels. This deviation is attributable to post-transcriptional regulatory mechanisms, variations in translation efficiency, and protein turnover rates. Transcripts are subject to stochastic fluctuations, whereas proteins integrate regulatory inputs over longer timescales and provide the functional effectors of cellular processes [43]. In this regard, proteomics complement qPCR by providing a more direct representation of the functional phenotype of cells. Consequently, the integration of these two approaches provides a more comprehensive understanding of the behavior of chondrocytes on biomaterial films.

In addition, despite the general similarities, the expression patterns of chondrocyte phenotype markers were different, even between cells growing on different biomaterials, supporting the global differences highlighted by UMAP.

Very interesting insights were obtained from the analysis of proteins involved in actin organization, cell adhesion, and cytoskeletal-nuclear remodeling. CFL and ARPC2 were upregulated in passaged chondrocytes, which had increased levels of F-actin (decreased levels of G/F-actin).

The role of CFL in passaged chondrocytes has not been clearly defined. CFL genes encode cofilins, a family of actin-depolymerizing factors present in chondrocytes. These factors influence the shape of cells, their interaction with the extracellular matrix, and the maintenance of the chondrocyte phenotype by regulating the actin cytoskeleton. One study found that knocking down CFL in passaged chondrocytes promoted actin polymerization and *Coll1* gene overexpression [44].

ARPC2 is a subunit of the Arp2/3 complex and a downstream effector of the Rho GTPase CDC42. CDC42 is crucial for regulating the actin cytoskeleton in cells. It acts as an actin nucleator, polymerizing the formation of new actin filaments. Some studies have shown that dedifferentiation in passaged chondrocytes is associated with RhoA induction, actin polymerization, and stress fiber (SF) formation. This establishes that actin polymerization is associated with chondrocyte dedifferentiation, while actin depolymerization is associated with chondrocyte redifferentiation [45].

Accordingly, both CFL and ARPC2 were both significantly overexpressed, primarily in P chondrocytes.

Conversely, the overexpression of adseverin, an actin severing and capping protein, was only observed in IS cells. Adseverin, a novel biomarker of chondrocyte phenotype, plays a role in regulating chondrocyte phenotype and function, preventing chondrocyte dedifferentiation and hypertrophy [46,47]. Although adseverin expression was downregulated in F and F+HA, the expression of actin biomarkers was significantly lower in cells cultured with biomaterials compared with P.

Dedifferentiated chondrocytes showed the overexpression of FN1 and ITGB1 and a shift in integrin complex composition, with ITGA3 upregulated and ITGA5 downregulated. These observations are consistent with the phenotypic shift during in vitro dedifferentiation [48,49]. ITGA3-ITGB1 overexpression is associated with both OA and a pro-inflammatory state. High levels of ITGA3 have been positively associated with osteoarthritis [50]. The pro-inflammatory state is mediated by the activation of the MAPK signaling cascade [51]. This result is consistent with the insight that we gained from the proteins associated with the dedifferentiation cluster and the pro-inflammatory markers.

Moreover, lamin B1 and CDC42 were overexpressed in P. CDC42 (cell division control protein 42), a small GTPase, is a member of the Rho GTPase family and is involved in chondrocyte differentiation. It regulates the formation of the F-actin cytoskeleton and stress fibers as well as cellular shape [52]. Lamin B1 is a structural protein located in the inner part of the nuclear lamina. It contributes to the integrity, stability, and function of the nucleus in chondrocytes. Interestingly, the overexpression of both proteins is associated with osteoarthritis (OA). In this condition, the overexpression of these two proteins is associated with dedifferentiation and cytoskeletal remodeling [53]. Their downregulation in F and F+HA (especially in F) suggests a potential role of biomaterials in promoting chondrocyte differentiation/redifferentiation.

Recently, Chen, Yu, Wen et al. [41] demonstrated a time-lapse atlas of chondrocyte dedifferentiation (P0–P8 passages), providing molecular details and identifying biomarkers associated with clinical chondrocyte evaluation. Using single-cell RNA sequencing data, they were able to identify four distinct cell subpopulations described by cluster-specific genes using Seurat unsupervised clustering [54]. Analysis of cluster distribution in each passage showed that pro and ecm markers were evenly distributed in differentiated P0 cells, while met markers started to join pro and ecm genes in early dedifferentiated chondrocytes (P2). Degradative genes were found to be descriptive of late stages of dedifferentiation (P4–P8). In addition, chondrocyte dedifferentiation was associated with metabolic stress and mitochondrial dysfunction, particularly related to reactive oxygen species (ROS) production. By pharmacologically manipulating mitochondrial stress signaling [55] to ameliorate the chondrocyte phenotype loss, the gain-of-function of early dedifferentiated cells was associated with the downregulation of the MAPK pathway [41]. The MAPK signaling pathway has been identified as a retrograde signaling pathway from the mitochondria to the nucleus during metabolic stress that can be activated by ROS [56]. Finally, the GO enrichment analysis is discussed.

The overexpressed DEPs of cells cultured on biomaterials showed an enrichment in biological processes involved in glycolysis (glycerol-3-phosphate metabolic process) (F and F+HA) and a deenrichment of GO terms associated with immune response (F), a signature already observed in P0 and at a very early stage of differentiation [41]. Nevertheless, a similar pattern was observed for P cells (Supplementary Table S8), with the exception of a significant deenrichment of the canonical Wnt pathway, which may be related to the low levels of SOX9 [57] and dedifferentiation through Wnt dysregulation [58]. With respect to Supplementary Figure S8, the most striking finding was the remarkable presence of GO terms associated with the tricarboxylic acid (TCA) cycle. Chondrocytes exist in a hypoxic environment in vivo, but evidence has shown that they are capable of using both anaerobic and aerobic metabolism to generate ATP [59], especially under some non-physiological conditions. This paradox may be explained by considering that the TCA cycle is an essential source of intermediary metabolites and energy to support cell survival and tissue repair [60]. Recently, Arnold et al. [61] discovered a non-canonical tricarboxylic acid (TCA) cycle in which acetyl-CoA is produced by ATP citrate lyase (ACL) from mitochondrially derived citrate, regenerating oxaloacetate in the cytoplasm. Oxaloacetate is converted to malate in a two-way reaction by malate dehydrogenase (MDH1), which is imported into the mitochondria by the mitochondrial citrate/malate antiporter SLC25A1, where it fuels oxaloacetate and citrate production via the canonical TCA cycle. The breakthrough finding was the involvement of the non-canonical TCA cycle in the regulation of cell identity [61,62,63]. Embryonic stem (ES) cells showed a higher degree of non-canonical TCA cycle activity compared with differentiated cells that switched to the traditional TCA cycle. Interestingly, the exit from naïve pluripotency requires engagement of the non-canonical cycle. By genetic manipulation of the ACL–SLC25A1–MDH1 axis, its involvement in the establishment of cell identity (undifferentiated-differentiated) has been demonstrated [61]. In light of these findings, the strong mitochondrial activity highlighted by GO enrichment analysis and the downregulation of MAPK pathway markers circumscribes a possible influence of biomaterials on mitochondrial metabolic activity and homeostasis, which may contribute to the maintenance of the adult differentiated phenotype observed in vitro. All in all, although this multidisciplinary approach expanded our biological understanding at the molecular level, a set of independent experiments must be designed to validate the hypothesis. Targeted LC-MS/MS proteomics, Western blot, and immunocytochemistry could be used to quantitatively validate protein levels. Additionally, functional assays of mitochondrial function (e.g., dynamic changes, endpoint assays, superoxide production) could support and expand our understanding of the biological processes responsible for changes in phenotype.

## 4. Materials and Methods

### 4.1. Reagents

Acetonitrile and acetic acid (99.8% *v*/*v*) were supplied by Sigma-Aldrich (St. Louis, MO, USA). Water was purified (0.055 uS/cm, TOC 1ppb) with a Purelab pulse + Flex ultra-pure water system (Elga Veolia, Milan, Italy). Iodoacetamide (IAA), dithiothreitol (DTT), urea, and trypsin were purchased from Sigma-Aldrich. Chitosan (ChitoClear^®^ Fg90 TM1874-CAS 9012-76-4, degree of deacetylation 95%; molecular weight by gel permeation chromatography 150–200 kDa; allergen free, water insoluble, soluble in acid media) was from Primex Ehf (Siglufjörður, Iceland). Hyaluronan sodium salt (50–60 kDa) was purchased from Primex Ehf (Siglufjörður, Iceland).

### 4.2. 2D Film Preparation

Chitosan powder was dispersed in an acetic acid solution (2% *v*/*v*) at the concentration of 6% *m*/*v*. The suspension was magnetically stirred for 24 h, then when the polymer was completely dissolved, raffinose pentahydrate (Sigma-Aldrich; St. Louis, MO, USA) was added as a rheological agent at a final concentration of 290 mmol/L and stirred for another 12 h. Bidimensional films were prepared by spreading out one milliliter of each solution onto a microscope slide (12 × 25 mm) to obtain a film with a uniform thickness of 0.25 mm and dried at 45 °C in a ventilated oven for 1 h. Finally, the polymeric hydrogel underwent ionotropic gelation to maintain its structure by exposition to a potassium hydroxide aqueous solution (1.5 mol/L) for 1 h. After gelation, hydrogels were washed twice in 80 mL of ultrapure water for 10 min to remove the gelling agent. Chitosan-hyaluronan scaffolds were prepared as described above by adding hyaluronan sodium salt at the concentration of 2% (*m*/*v*) into the initial chitosan solution.

### 4.3. Cell Isolation and Cell Culture

Articular cartilage was obtained from 12 fetlock joints of adult horses (5–8 years old) without any distinction on sex or breed, slaughtered for human consumption in a slaughterhouse, and delivered to the laboratory within 1 h in controlled conditions [6,35,64]. Briefly, cartilage explants were finely diced under sterile conditions, and then the slices were pooled (4 joints/experiment) and washed several times in phosphate-buffered saline (PBS 1X). The tissue digestion was carried out by pre-incubation of tissue in 0.1% pronase (Merck; Darmstadt, Germany) in DMEM for 1 h at 37 °C, and then by final digestion in 0.2% collagenase type IA (Merck; Darmstadt, Germany) in DMEM for 2 h at 37 °C. The digested tissue was filtered through 100 µm and 20 µm nylon filters, and the cellular suspension was centrifuged at 400× *g* for 10 min. The pellet was washed several times with DMEM (4.5 g/L glucose; 25 mmol/L HEPES) containing 10% fetal calf serum (FCS), 100 U/mL penicillin, and 0.1 mg/mL streptomycin. The chondrocytes were counted using a hemocytometer, and the cell viability (>95%) was assessed by the Trypan Blue (0.1%) exclusion assay. Freshly isolated chondrocytes (P0) were seeded (2 × 10^4^ cells/cm^2^) in triplicates and cultured for 2 weeks (P1: first passage culture) in adhesion to:

Tissue culture dish (21 cm^2^) (Sarstedt, Nümbrecht, Germany) (P: plastic);
Tissue culture dish (21 cm^2^) covered with film of chitosan (F);
Tissue culture dish (21 cm^2^) covered with film of chitosan-hyaluronic acid (F+HA).

The primary chondrocytes were cultured in medium DMEM 4.5 g/L glucose (Sigma-Aldrich; St. Louis, MO, USA) with 10% FBS and incubated in a thermostat at 37 °C and 5% of CO_2_ for 2 weeks; three independent experiments were performed. The medium was changed every three days. Finally, primary chondrocytes grown in the three different 2D cultures were detached with 0.25% trypsin–0.02% EDTA (Merck; Darmstadt, Germany) and centrifuged to obtain the pellets used for subsequent analysis.

### 4.4. Morphological Analysis

Histochemical evaluations were performed in chondrocytes grown on tissue culture dishes and biomaterial films for 2 weeks. After fixing with 4% paraformaldehyde for 40 min at room temperature, the samples were dehydrated in ascending grades of ethanol, cleared, embedded in paraffin, and sectioned at a thickness of 5 µm. The sections were stained with hematoxylin and eosin (H&E).

### 4.5. Total RNA Extraction

The total RNA of each sample was extracted using TRIreagent (Invitrogen; Carlsbad, CA, USA) according to the manufacturer’s instructions. Purity and concentration were assessed by UV spectrophotometry at 260/280 nm and 260/230 nm, respectively. RNA integrity and quality were assessed using an Agilent Bioanalyzer 2100 and RNA 6000 Labchip Kit (Agilent Technologies; Santa Clara, CA, USA).

### 4.6. Reverse Transcription (RT)

RNA samples were DNAse-treated (Sigma-Aldrich; St. Louis, MO, USA) prior to cDNA synthesis. Total RNA (1 μg/20 μL) was converted to cDNA by a High-Capacity cDNA Reverse Transcription Kit (Applied Biosystems; Carlsbad, CA, USA) according to the manufacturer’s instructions.

### 4.7. Quantification of mRNA by Real-Time PCR (qPCR)

Five ng of each sample was used as a template for real-time quantitative PCR (qPCR) performed using a StepOne thermocycler (Applied Biosystems, StepOne software version 2.3). The cDNA was amplified in triplicates with Power Up SYBR Green Master Mix (Applied Biosystems, USA) along with specific sets of primers at 300 or 500 nmol/L (Supplementary Table S1). The GAPDH gene was selected among other tested reference genes (i.e., 18S, β-2MG, and HPRT) as the endogenous control due to minimal intra-/inter-assay variation. A no-RT control and a no-template control (NTC) were included in each experiment. Data were analyzed according to the 2^−ΔΔCt^ method [29,30] in which the expression levels of each gene were normalized to the GAPDH cDNA amount and expressed as relative quantities (RQs).

### 4.8. Protein Extraction and Sample Preparation for LC-MS/MS

Total proteins were extracted from cell pellets using 8 mol/L urea in 100 mmol/L Tris-HCl pH 8 lysis buffer and sonicated at 4 °C for 5 cycles of 30 s ON/OFF. Extracts were clarified by centrifugation and proteins were quantified using the BCA assay. For each biological replicate, an amount of 50 µg was reduced by 10 mmol/L TCEP, alkylated by 40 mmol/L chloroacetamide, digested by Lys-C, and then by trypsin in a 1:50 (enzyme:protein) ratio overnight. Peptides were desalted on a custom-built StageTip C18 and analyzed by MS.

### 4.9. Shotgun LC-MS/MS Proteomics

A total of 2 µL of peptide mixture for each biological replicate was injected as technical replicates (n = 2) on an nLC–ESI–MS-MS quadrupole Orbitrap QExactive-HF mass spectrometer (Thermo Fisher Scientific; San José, CA, USA). Peptide separation was achieved on a linear gradient from 100% solvent A (2% ACN, 0.1% formic acid) to 30% solvent B (80% acetonitrile, 0.1% formic acid) over 107 min and from 30 to 60% solvent B in 5 min at a flow rate of 0.25 µL/min on an UHPLC Easy-nLC 1000 (Thermo Scientific) connected to a 23 cm fused-silica emitter with a 75 µm inner diameter (New Objective; Littleton, MA, USA), packed in-house with ReproSil-Pur C18-AQ 1.9 µm beads (Dr Maisch Gmbh; Ammerbuch, Germany) using a high-pressure bomb loader (Proxeon; Odense, Denmark). MS data were acquired using a data-dependent top 20 acquisition with HCD fragmentation. Survey full scan MS spectra (300–1650 Th) were acquired using Orbitrap mass analyzer with a resolution equal to 60,000, automated gain control (AGC) 3 × 10^6^ (targets), and an injection time (IT) of 20 ms. For HCD spectra, the resolution was set to 15,000 at *m*/*z* 200, AGC 1 × 10^5^ (targets), IT 80 ms, normalized collision energy (NCE) of 28%, isolation width 1.2 *m*/*z,* and a dynamic exclusion of 20 s. To control potential background signals or carry-over phenomena, each sample run was separated by five blank injections. The mass spectrometry raw files were processed using Skyline (version 24.1) for peptide and protein identification, with the LFQ algorithm enabled. Database identification was performed against the Uniprot *Equus caballus* proteome (October 2024). Search parameters included cysteine carbamidomethylation as a fixed modification and N-terminal acetylation and methionine oxidation as variable modifications. Peptide spectrum matching (PSM) was performed looking for precursors with charges between +2 and +7. Up to two missed cleavages were allowed for specific protease digestion. The false discovery rate (FDR) for protein identification was set to 0.05 and the minimum peptide length was fixed at 7. The protein peak integrations were manually inspected. Retention time consistency, chromatographic landscape, and signal intensity were used to cross-validate the automated integration results. Peptides with at least 3 y and 3 b ions were retained. Peptide identity was confirmed based on fragments coelution and retention time consistency between each replicate; only proteins found in both replicates were considered for the statistical analysis.

### 4.10. Gene Expression and Untargeted Proteomics Statistical Analysis

For gene expression, data were analyzed with one-way analysis of variance (ANOVA) using the SPSS v.28 for Windows (IBM^®^ SPSS^®^ Statistics, IBM, Armonk, NY, USA) with subsequent Dunnett’s multiple comparison test (GraphPad Prism 7.0; La Jolla, CA, USA). Experimental data were presented as the mean ± standard deviation. The figures show the statistical differences in gene expression between the groups, which were considered significant at *p*-value < 0.05 (*), *p*-value < 0.01 (**), and *p*-value < 0.001 (***).

Preprocessing of untargeted proteomics data was performed using the R package “protti” (version 0.9.0). For statistical evaluation, only proteins that were identified by at least three peptides were considered. LC-MS/MS peak areas were log_2_ and median normalized (peak areas for each run were normalized to the median of all runs). Normalized data were filtered to remove all proteins with a coefficient of variation (CV%) greater than or equal to 25% (Supplementary Figure S1) in at least one of the experimental conditions. Differential protein abundance was calculated using a moderate t-test function embedded in “protti”. Prior to calculation, the distributional assumptions (i.e., normality) of the test were verified. To calculate the fold change, an independent pairwise comparison was made for a total of six comparison: IS vs. P, IS vs. F, IS vs. F+HA, P vs. F, P vs. F+HA, F vs. F+HA, according to the formula for independent pairwise comparison for *k* = 4 conditions:

*k*(*k* − 1)/2

The adjusted *p* value (Benjamini–Hochberg) and the fold change (fc) thresholds were set at an adj *p* value < 0.01 and fc >|1|. Uniform manifold approximation and projection (UMAP) was used to evaluate the global profile of the experimental conditions according to the normalized LC-MS/MS peak areas (mean centered and scaled to standard deviation) of detected proteins using the “umap” R package. 3D UMAP plots were generated using the R package “plotly”. Volcano plots were used to visualize between the pairwise comparisons, the fold change, and the statistical significance of the protein markers (COL1 (A1 and A2), COL2, ACAN, SOX9, CFL (1 and 2), ARPC2, SCIN, FN1, ITGA5, ITGA3, ITGB1, LMNB1, CDC42) associated with the phenotype of differentiated and dedifferentiated chondrocytes (Supplementary Tables S2–S5). A PCA was calculated using the R packages “stats” and “factoextra” to evaluate the normalized LC-MS/MS peak areas (mean centered and scaled to standard deviation) of protein markers associated with chondrocyte dedifferentiation stages (Supplementary Table S6). The selection of biomarkers was based on the chondrocyte dedifferentiation model established by Chen, Yu, Wen et al. [31]. Finally, the “protti” Gene Ontology (GO) enrichment analysis function was performed using the proteins overexpressed in each experimental condition (biological process GO terms) (Supplementary Tables S7–S10).

## 5. Conclusions

Mass spectrometry was used to study the early dedifferentiation process that occurs during in vitro cell adhesion and expansion. This 2D model was also used to evaluate chondrocyte expansion on different substrates including plastic, chitosan, and hyaluronic acid-based chitosan, which are considered suitable scaffolds for cartilage engineering. Our results showed that the 2D film culture system allowed the chondrocytes to maintain a round-like morphology within the first two weeks of culture (passage P1) in contrast to early dedifferentiation in tissue culture dishes where the chondrocytes assumed a fibroblast-like shape. Furthermore, these results were also associated with the maintenance of chondrocyte differentiation marker expressions such as type II collagen, aggrecan, *Sox9* and *Coll6*. Mass spectrometry data revealed a different proteomic profile of cells grown on different biomaterials, suggesting dynamic cell–biomaterial interactions promising to support chondrogenesis. The proteomic analysis showed strong consistency with the state-of-the-art of the biological mechanisms of differentiation/dedifferentiation, generating new hypotheses about the effect of 2D films on mitochondrial activity and homeostasis.

Finally, since our results showed that critical biological perturbations occur in the first passage of in vitro expansion, this in vitro model may allow us to better define when and how chondrocytes can withstand before reaching a dedifferentiated state that does not allow for restoration of the naïve phenotype, thus excluding these dedifferentiated cells from use in cell therapies or tissue engineering.

Despite testing the chondro-inductive ability of different biomaterials, proteomic analysis has provided thorough insights into the complex relationships between chondrocyte cells and new materials for tissue bioengineering. This analysis offers a novel, general perspective on the molecular changes that occur and the potential to apply them as new markers of chondrocyte differentiation.

Finally, these results could also have an important impact in future studies on identifying potential treatments for the redifferentiation.

## Data Availability

The raw data supporting the conclusions of this article will be made available by the authors on request.

## Supplementary Materials

The following supporting information can be downloaded at: https://www.mdpi.com/article/10.3390/ijms262110291/s1.
