# Supplementary material for: 2D Chitosan-Based Films: A Proteomic Mass Spectrometry Study of Chondrocyte Phenotype as a Function of Cell–Biomaterial Interactions
Source: Int J Mol Sci. 2025 Oct 22;26(21):10291. doi: 10.3390/ijms262110291 (PMC12608676; doi:10.3390/ijms262110291)
Supplement: Supplementary file 1 [file ijms-26-10291-s001.zip › Supplementary Figures S1-S8.pdf]

Supplementary Materials

2D chitosan-based films: a proteomic mass spectrometry study of chondrocyte differentiation processes as a function of cell-biomaterial interactions

Alessandro Zaccarelli[1], Roberta Saleri[2], Elena De Angelis[2], Francesca Ravanetti[2]*, Attilio Corradi[2] Paolo Borghetti[2]

[1] Food and Drug Department, University of Parma, Viale delle Scienze 17/a, 43124, Parma, Italy.

[2] Department of Veterinary Science, University of Parma, Strada del Taglio 10, 43126, Parma, Italy.

* Corresponding Author: Francesca Ravanetti

Phone: +39 0521 032647

Fax: +39 0521 032795

E-mail: francesca.ravanetti@unipr.it

Technical and combined coefficients of variation (CV%) between experimental conditions in shotgun proteomics.

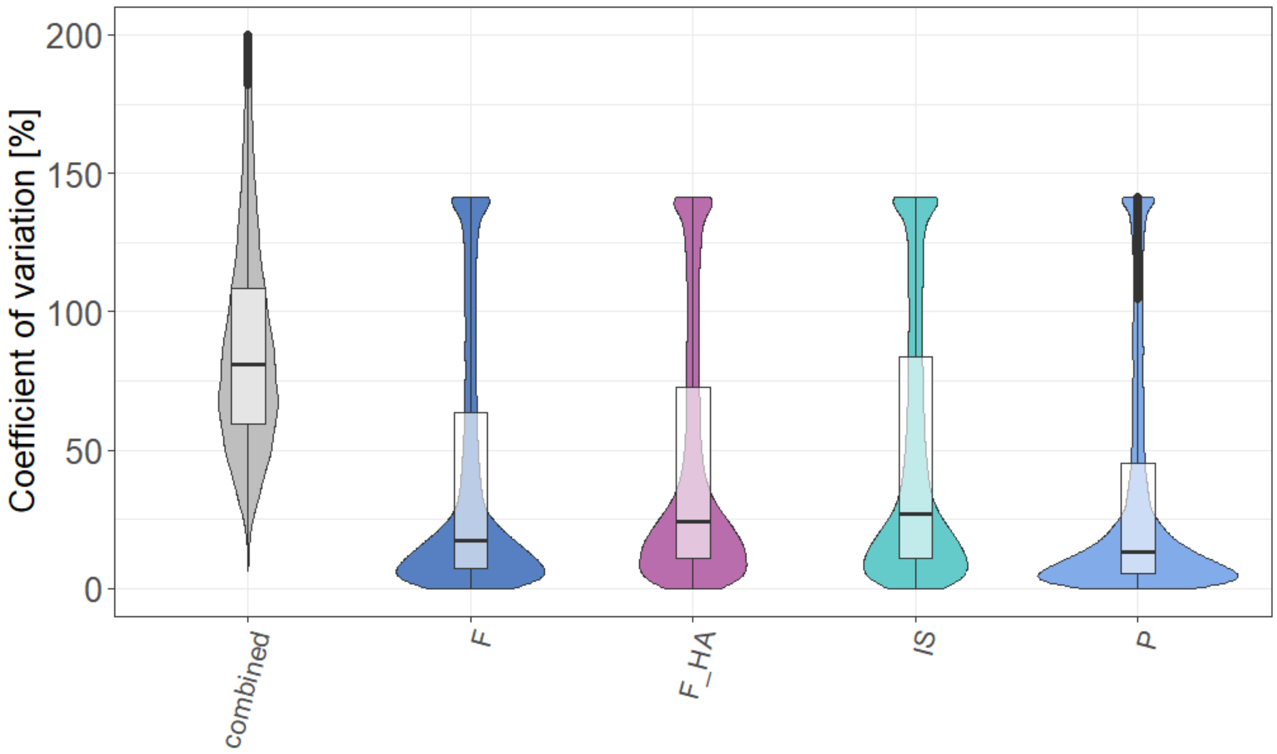

Figure S1. Violin plot for the comparison of technical and combined CV % between experimental conditions.

Gene expression analysis of cartilage markers.

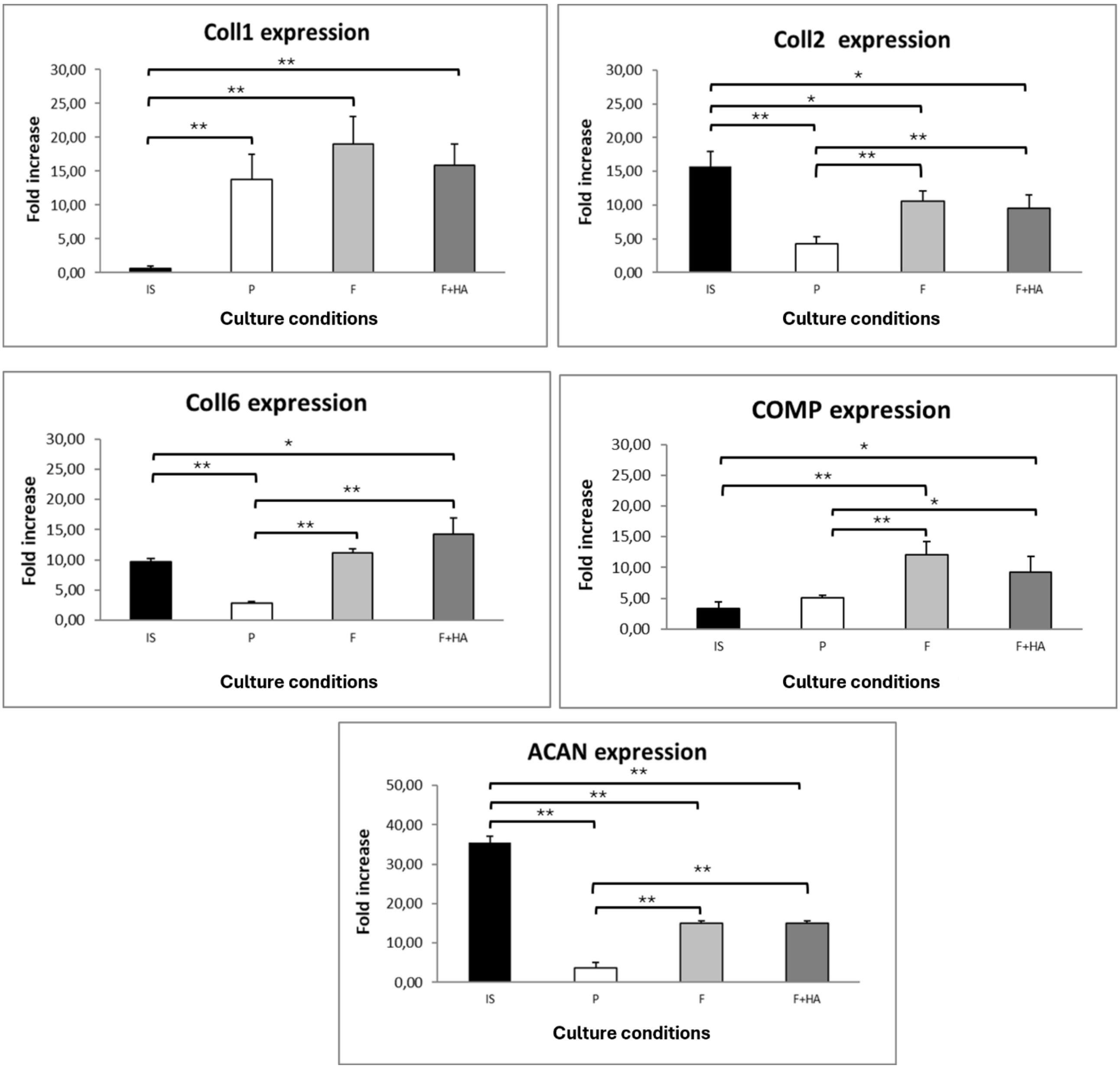

Figure S2. Gene expression of *Coll1*, *Coll2, Acan* , *Coll6* and *COMP* in freshly isolated chondrocytes (IS), cultured in adhesion on tissue culture dish (P), on chitosan film (F), and on chitosan film+hyaluronic acid (F+HA) for 2 weeks. *: p value < 0.05; **: p value < 0.01.

Gene expression analysis of transcription factors.

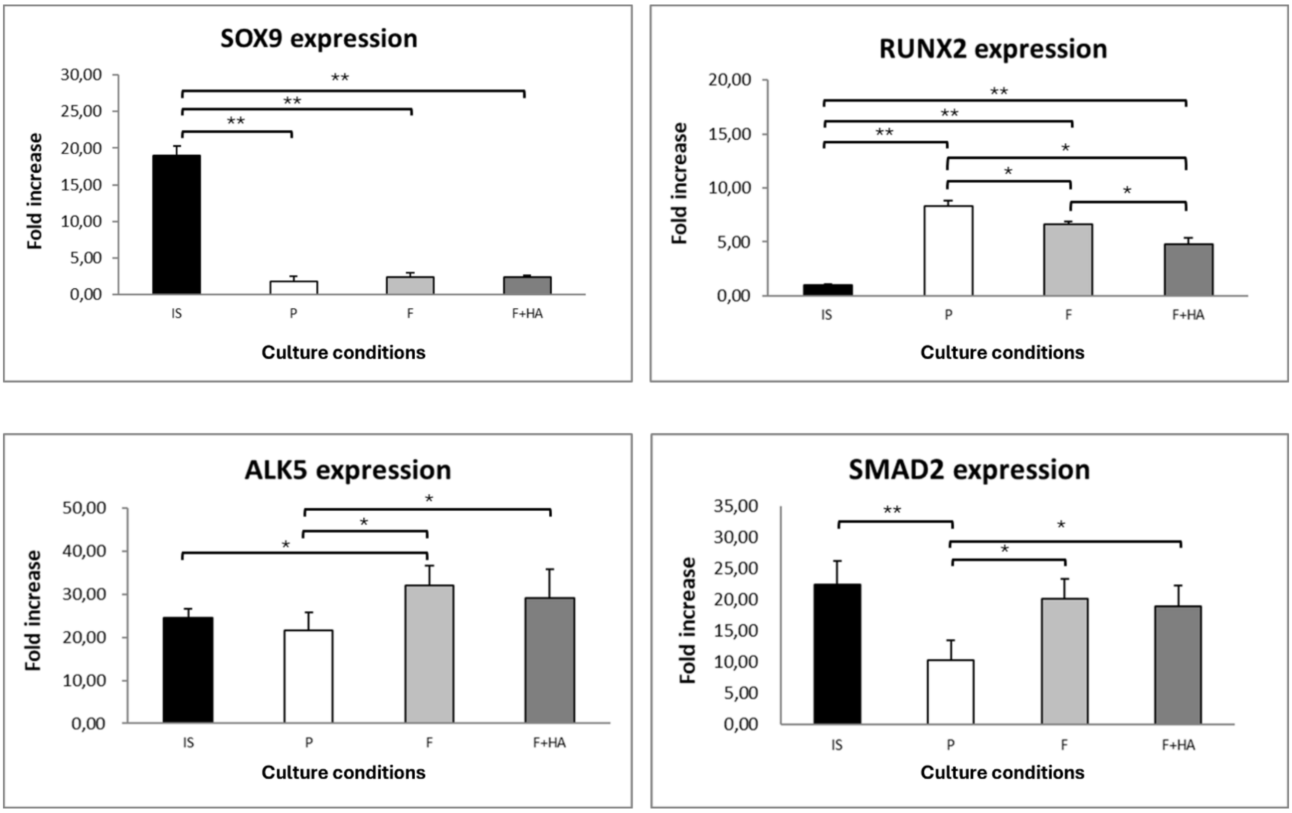

Figure S3. Gene expression of *Sox9*, *RUNX2*, *ALK5* and *SMAD2* in freshly isolated chondrocytes (IS), cultured in adhesion on tissue culture dish (P), on chitosan film (F), and on chitosan film+hyaluronic acid (F+HA) for 2 weeks. *: p value < 0.05; **: p value < 0.01.

Volcano plots for pairwise comparison in proteomics.

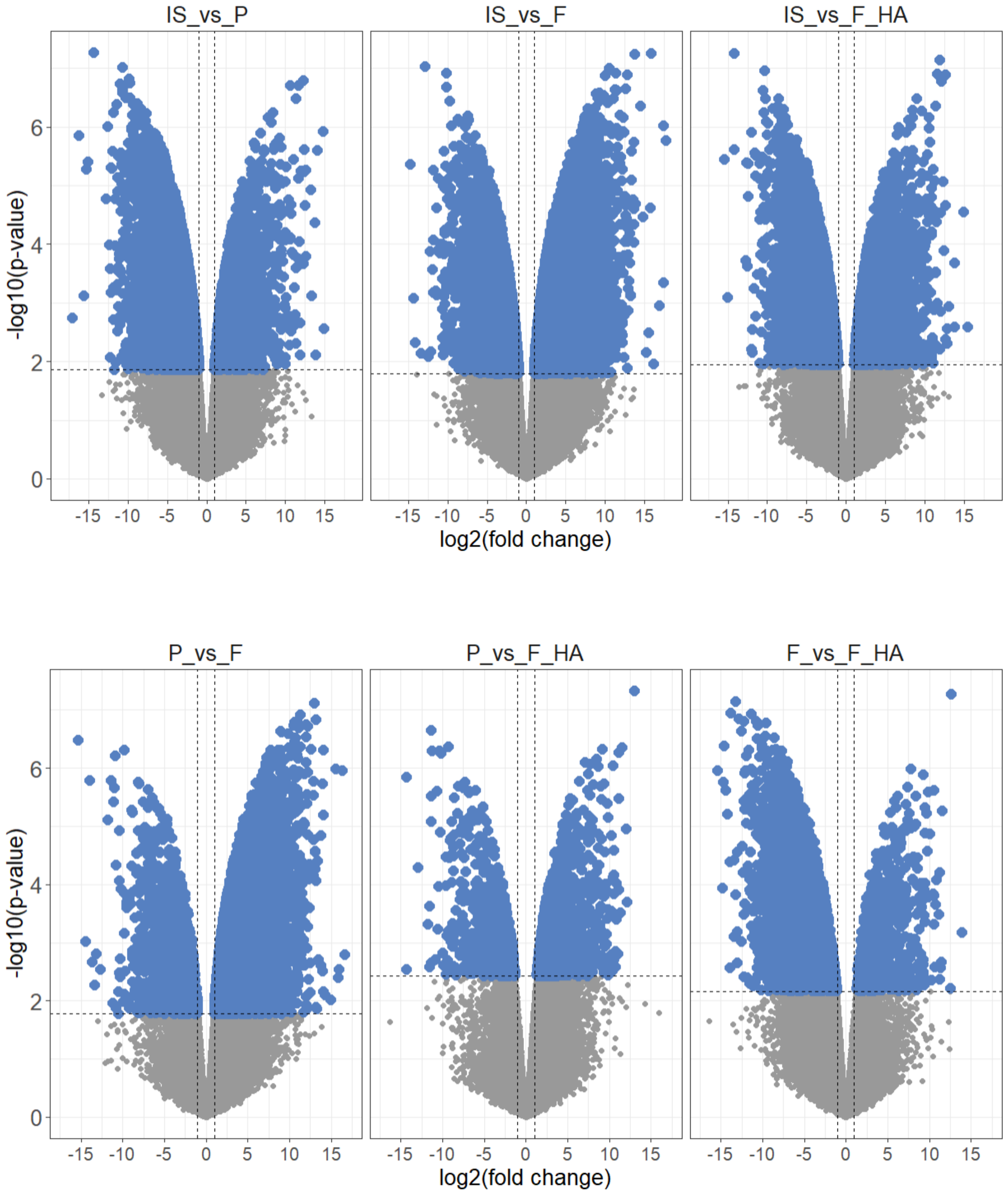

Figure S4. Fold change and statistical significance in pairwise comparison. Proteins are represented by all the precursor peptides identified by shotgun proteomics.

Differential expression of actin organization markers.

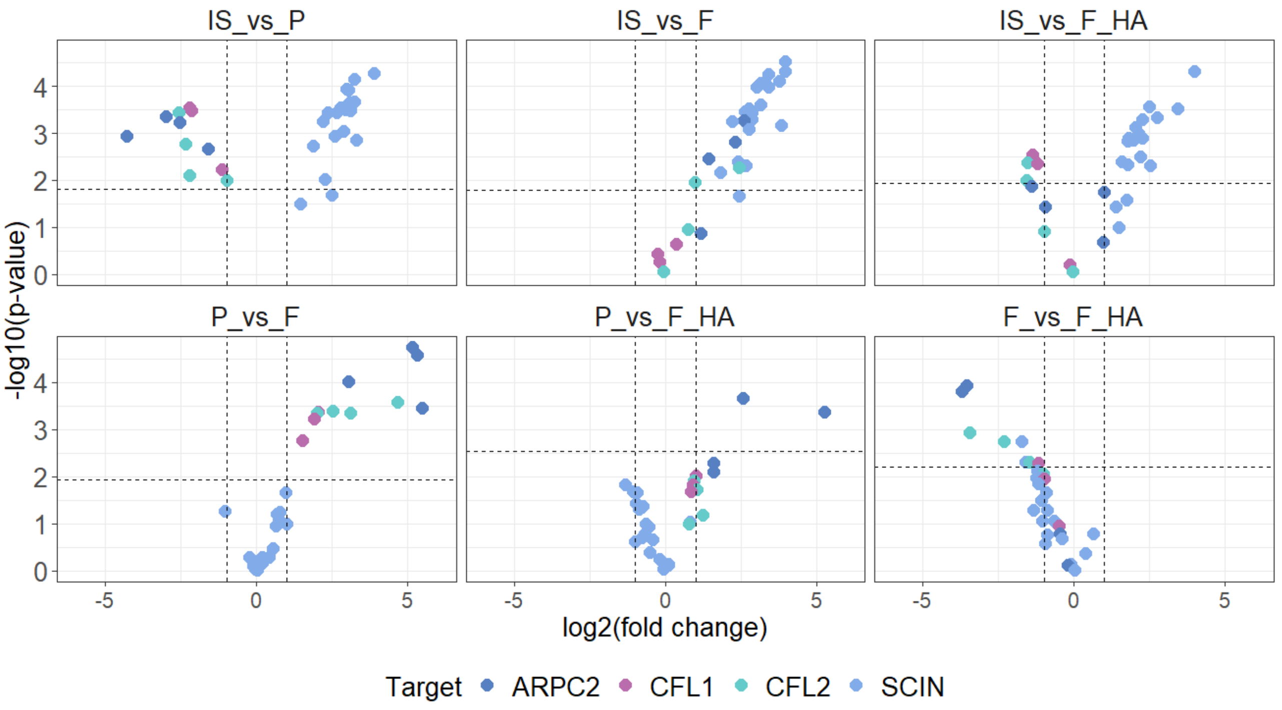

Figure S5. Fold change and statistical significance of actin organization protein markers in pairwise comparison. Proteins are represented by all the precursor peptides identified by shotgun proteomics.

Differential expression of cell adhesion markers.

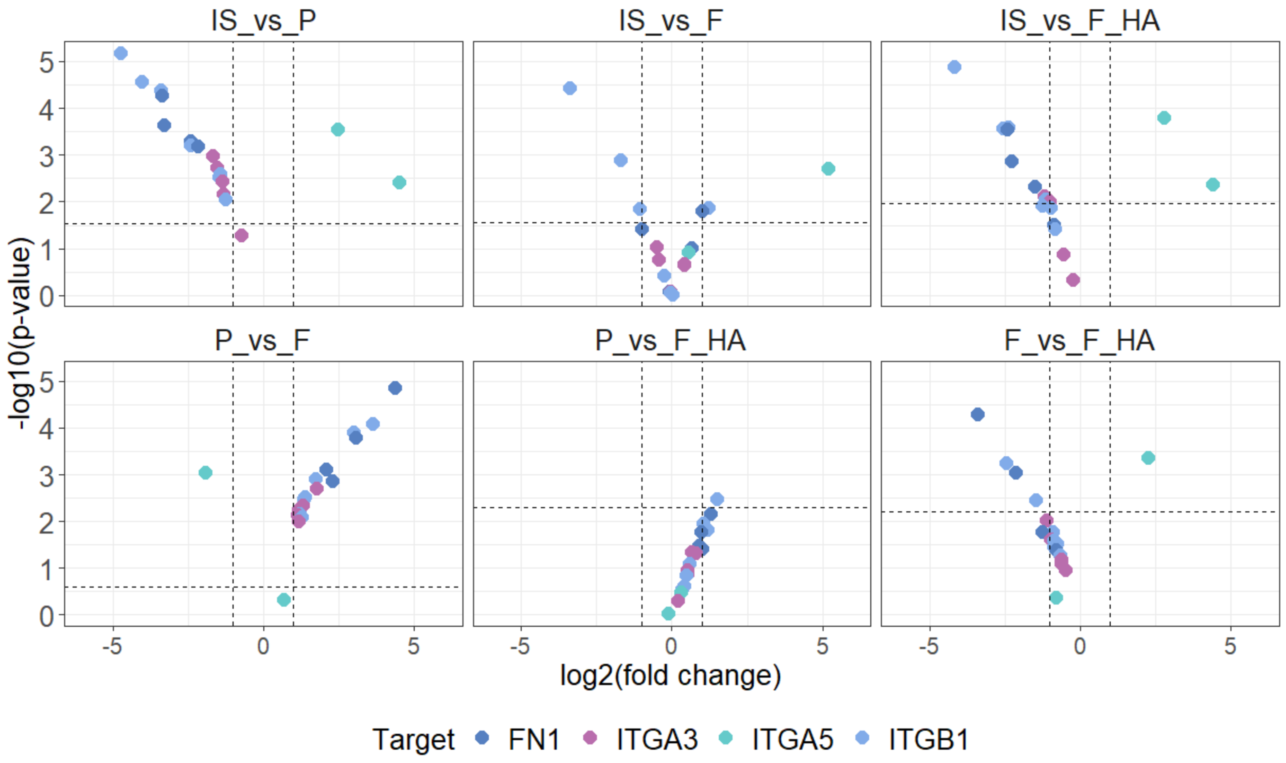

Figure S6. Fold change and statistical significance of cell adhesion protein markers in pairwise comparison. Proteins are represented by all the precursor peptides identified by shotgun proteomics.

Differential expression of cytoskeletal-nuclear remodeling markers.

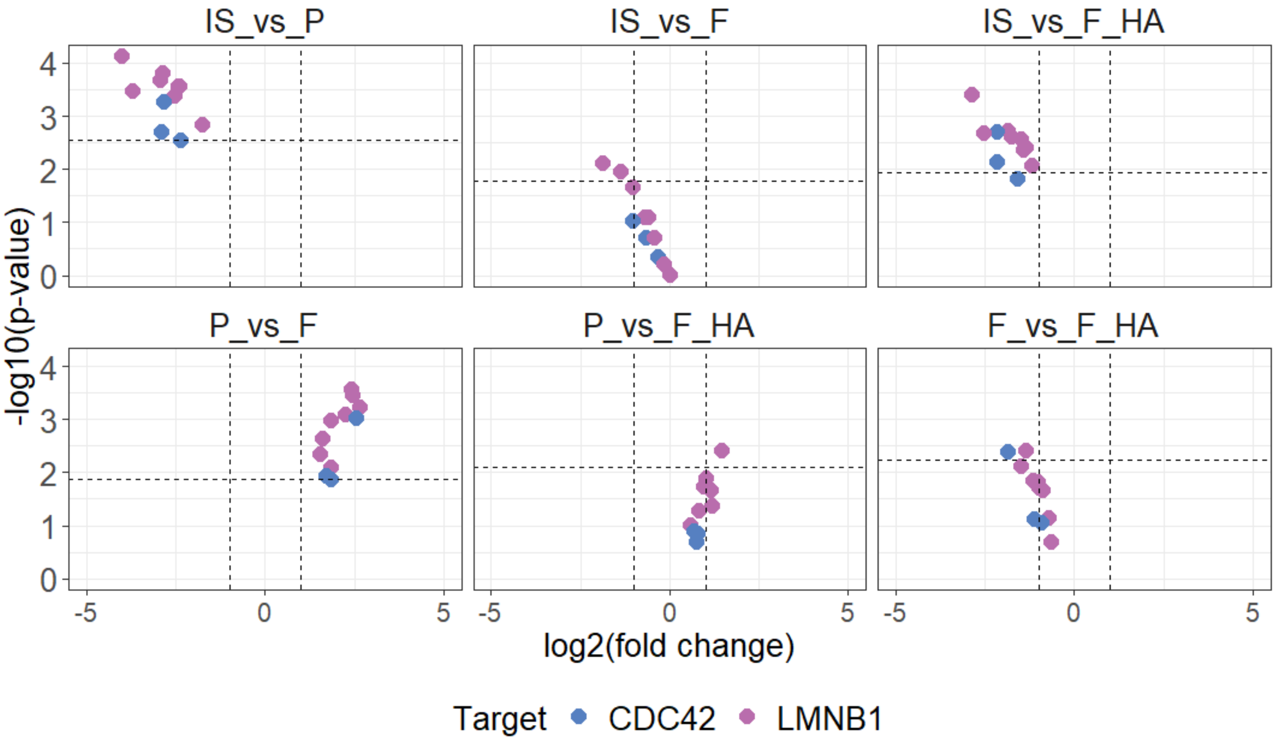

Figure S7. Fold change and statistical significance of cytoskeletal-nuclear remodeling markers in pairwise comparison. Proteins are represented by all the precursor peptides identified by shotgun proteomics.

Gene ontology enrichment analysis of DEPs in cells grown on biomaterials.

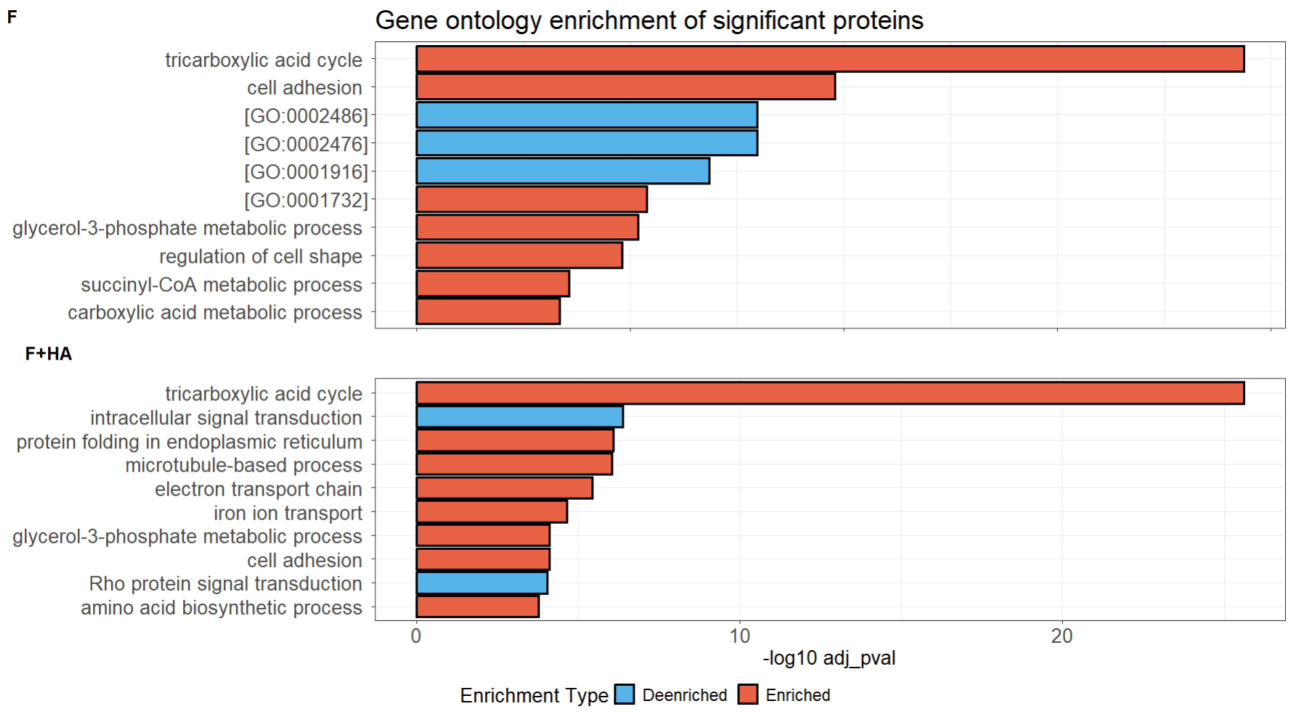

Figure S8. GO enrichment analysis of F and F+HA significant proteins on GO biological process annotation set. GO0002486/2476/1916 terms belong to immune system response, while GO0001732 is related to translation initiation.
